# Using Small-Area Estimation Method to Calculate County-Level Prevalence of Obesity in Mississippi, 2007-2009

**Published:** 2011-06-15

**Authors:** Zhen Zhang, Lei Zhang, Alan Penman, Warren May

**Affiliations:** Center of Biostatistics and Bioinformatics, University of Mississippi Medical Center; Mississippi State Department of Health, Jackson, Mississippi; University of Mississippi Medical Center, Jackson, Mississippi; University of Mississippi Medical Center, Jackson, Mississippi

## Abstract

**Introduction:**

Obesity is one of Mississippi's pressing public health problems. Since 2005, the state has ranked first in the nation in adult obesity prevalence. For authorities to take targeted action against the obesity epidemic, counties, regions, and subpopulations that are most affected by obesity need to be identified. The objective of this study was to assess the scope, socioeconomic and geographic characteristics, and temporal trends of the obesity epidemic in Mississippi.

**Methods:**

Using 2007-2009 Mississippi Behavioral Risk Factor Surveillance System data and auxiliary data, we applied a small-area estimation method to estimate county-level obesity prevalence in 2007 through 2009, to assess the association between obesity and socioeconomic factors and to evaluate temporal trends. We determined geographic patterns by mapping obesity prevalence. We appraised the precision of estimates by the width of 95% confidence intervals, and we validated our small-area estimates by comparing them with direct estimates.

**Results:**

In 2009, the county prevalence of obesity ranged from 30.5% to 44.2%. Counties with the highest prevalence of obesity were in the Delta region and along the Mississippi River. The obesity prevalence increased from 2007 through 2009. Age, sex, race, education, and employment status were associated with obesity.

**Conclusion:**

The 2009 obesity prevalence in all Mississippi counties was substantially higher than the national average and differed by geography and race. Although urgent intervention measures are needed in the entire state, policies and programs giving higher priority to higher-risk areas and subpopulations identified by this study may be better strategies.

## Introduction

In the past 25 years, the prevalence of obesity, defined as a body mass index (BMI) of at least 30 kg/m^2^, has increased dramatically in the United States; more than one-third of adults are now obese (1-4). Mississippi has ranked first in the nation in obesity prevalence since 2005. Between 1995 and 2009, the prevalence of obesity in Mississippi increased substantially, from 19.5% to 35.4% (5), and there is no indication that this upward trend will level off soon. Obesity is associated with heart disease, diabetes, cancer, asthma, arthritis, stroke, and premature death (6,7). Consequently, obesity and its associated health problems have a substantial social and economic effect (6,8). According to the Mississippi State Department of Health (9), obesity is one of the state's most pressing public health problems. The high and increasing rate of diabetes in the state is highly correlated with the increasing rate of obesity (9,10). The highest obesity rates should be identified for priority intervention both because of limited resources and because obesity control programs may be more effective when tailored according to specific community needs (11,12).

Socioeconomic characteristics, such as age, sex, race, income, and education, and community factors such as average per capita income and percentage of labor-intensive workers are associated with obesity (3,13). In an effort to establish the association of diabetes with obesity, Centers for Disease Control and Prevention (CDC) produced 2007 county-level obesity prevalence estimates for 3,141 US counties by using Bayesian multilevel modeling (10) but did not analyze geographic and socioeconomic characteristics of the obesity epidemic for the states. To our knowledge, no studies have focused on providing up-to-date small-area health statistics and related information in Mississippi for prevention and intervention purposes. In this study, we sought to produce reliable county-level estimates of obesity prevalence for all Mississippi counties, identify geographic heterogeneity and temporal trends, and evaluate associations between obesity and socioeconomic factors. This information would enable the development of appropriate obesity prevention policies and community interventions.

## Methods

### Data sources

Established by CDC, the Behavioral Risk Factor Surveillance System (BRFSS) is an ongoing state-based surveillance system tracking health conditions and risk behaviors among noninstitutionalized adults aged 18 years or older in the United States. The design of BRFSS aims at generating reliable prevalence estimates at the state level or for large metropolitan statistical areas. Sample size for counties are usually too small for making direct inferences with satisfactory precision. CDC suggests that a sample size of at least 300 is necessary for direct estimation (14,15). To overcome the limitation of small sample sizes, we developed a 2-step estimation method. This method belongs to a family of small-area estimation techniques that includes varied approaches for making inferences about geographic or social subdomains of the survey domain. The power of the small-area estimation method resides on its ability to borrow strength from multiple sources of data — data collected at other times, or in related areas, or both to increase the effective sample size and thus achieve adequate precision. Among current small-area estimation methods, the Hierarchical Bayes approach and generalized linear mixed models have been primary choices (16-18). We used a generalized linear mixed model in this study. We incorporated a generalized linear mixed model and traditional synthetic methods to meet the small-area estimation needs in this study.

We acquired individual-level BRFSS data for Mississippi for 2007 through 2009. Variables included in the analytical dataset were self-reported height and weight and demographic and socioeconomic variables, including age, race, sex, education level, employment status, annual household income, and marital status. We used the Federal Information Processing Standard code as the location variable for county. We categorized BMI as a binary variable, obesity (obesity = 0 if BMI <30 kg/m^2^, obesity = 1 if BMI ≥30 kg/m^2^). In the original Mississippi BRFSS data, age is a continuous variable. Through initial exploratory data analysis, we found a quadratic relationship between BMI and age. According to the shape of the quadratic regression line, we categorized age into 4 groups: 18 to 29, 30 to 44, 45 to 64, and 65 years or older. We categorized education as less than a bachelor's degree and bachelor's degree or higher. For employment status, we grouped employed for wages and self-employed as "employed," and grouped unemployed temporarily, those unable to work, students, retired people, and homemakers as "unemployed."

The exclusion criteria were 1) missing Federal Information Processing Standard code information (n = 39); 2) missing BMI values or biologically unlikely BMI values (BMI <12 kg/m^2^ or BMI >70 kg/m^2^) (n = 1,013); 3) pregnant (n = 138); 4) races other than black and white (n = 383). Taking into consideration that in Mississippi 98.5% of the population belongs to 1 of 2 categories, black alone or combined and white alone or combined (19), we excluded the small portion (1.5%) of respondents of other races to facilitate small-area estimation. Respondents who chose black alone or black and other race(s) were categorized as black alone or combined; respondents who chose white alone or white with race(s) other than black were categorized as being in the white alone or combined category. The final analytical dataset of 2007-2009 BRFSS had 25,046 observations.

The data sources for auxiliary information were the 2000 US Census (19) and the US Department of Agriculture Economic Research Service (20). We used the auxiliary information to construct predictor variables for statistical modeling. Auxiliary covariates included county-specific sociodemographic factors such as county population composition of age, sex, race, educational achievement, county unemployment rate, rural-urban continuum characteristics, economic dependence indicators, and poverty indicator. Corresponding to the 4 age categories at the individual level, the auxiliary dataset included 4 age variables, which are the percentages of each age group 18 years or older in the county population.

### Estimation method

Modified on the basis of the multilevel logistic regression model of Li and colleagues (13), our 2-step small-area estimation method is a combination of hierarchical modeling and synthetic estimation techniques. This mixed model produces a set of fixed-effect parameter estimates, which are general to all counties, and a set of random-effect parameter estimates, which are county-specific values. First, we identified the independent variables that were significantly associated with obesity by fitting a generalized linear mixed model with the relevant variables, both individual-level and county-level. We used backward elimination to prune the model. To further improve predictability, we set the selection criterion for retaining a variable or interaction term in the model at *α* = 0.1 (21). In the final model, fixed effects included the following variables: age, sex, race, education level, employment status, survey year, and county-level average per capita annual household income. Random effects included individual-level variables age, sex, race, education level, and survey year. We also calculated odds ratios for associated socioeconomic factors in this step.

In the second step of the estimation, we applied a synthetic technique to link the model-generated parameter estimates to the county-specific characteristics to produce county-level estimates. This way, the strength borrowed through the model is realized for each county. Because random effects were not significant, we used only the fixed-effect values in the calculation. However, following the recommendation of Binder (22), Jiang (23), and Jia et al (24), we kept the random-effects component in the model to improve estimation for fixed effects and to enhance the proper selection of variables (Appendix). We conducted all analyses by using SAS version 9.2 (SAS Institute Inc, Cary, North Carolina) to account for the complex sampling design. We grouped the county-level obesity prevalence estimates into quartiles to examine geographic patterns. We produced the obesity map by using ArcGIS 9.2 (ESRI, Redlands, California).

We assessed the precision of our estimates by using the width of 95% confidence intervals (CIs). Following the example of another study (25), we examined correlations and mean absolute differences between model estimates and internal standards: direct estimates of the state-level obesity rate for 2007 through 2009, and direct estimates for age, sex, and race subgroups. The sample sizes for the 4 age subgroups, 2 sex subgroups, and 2 race subgroups were large enough for reliable direct estimation (range, 663-7,305). Taking into consideration the complex sampling design of BRFSS, we used SAS PROC SURVEYLOGISTIC procedure (SAS Institute, Inc, Cary, North Carolina) for direct estimations to account for the weight of each respondent.

## Results

**Figure 1:**
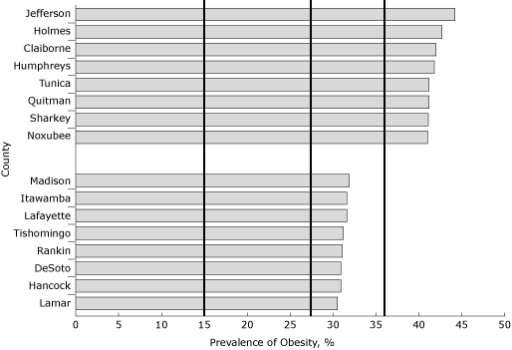
2009 Mississippi County prevalence of obesity, Behavioral Risk Factor Surveillance System (BRFSS) data. Ten percent of the counties with the lowest and highest obesity rates are shown. The vertical line at 15% indicates the *Healthy People 2010* goal for obesity; vertical lines at 27.1%, and 35.4% indicate the 2009 national and state averages, respectively. Obesity is defined as a BMI  ≥30.0 kg/m^2^ based on 2009 BRFSS self-reported data.

The state obesity prevalence increased significantly, from 32.5% in 2007 to 33.4% in 2008 and 35.4% in 2009. The prevalence estimates of all 82 counties were higher in 2009 than in 2007 (Tables 1 and 2). In 2009, all counties had prevalence of obesity greater than 30%. In 49 counties, the prevalence of obesity was at 35% or higher, and in 12 counties, was at least 40% or higher. Compared with the *Healthy People 2010* goal and with the 2009 national average, all of the county estimates were substantially higher (Figure 1).

**Table:** 

**County**	**2009 Prevalence of Obesity, %**	***Healthy People 2010* Goal, %**	**2009 National Average, %**	**2009 Mississippi State Average, %**
**Lowest 10%**		15.0	27.1	35.4
Lamar	30.5			
Hancock	30.9			
DeSoto	30.9			
Rankin	31.1			
Tishomingo	31.2			
Lafayette	31.6			
Itawamba	31.6			
Madison	31.9			
**Highest 10%**				
Noxubee	41.0			
Sharkey	41.1			
Quitman	41.2			
Tunica	41.2			
Humphreys	41.8			
Claiborne	42.0			
Holmes	42.7			
Jefferson	44.2			

**Figure 2:**
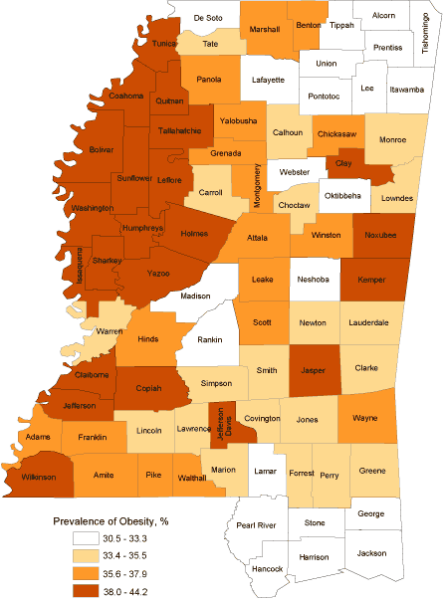
2009 Mississippi county prevalence of obesity map (in quartiles),  Behavioral Risk Factor Surveillance System (BRFSS) data. Lighter colors represent lower obesity rate, and darker colors represent higher obesity rate. Obesity is defined as BMI ≥30.0 kg/m2 based on 2009 BRFSS self-reported data.

There was considerable geographic variation in obesity prevalence among Mississippi counties. The difference between Jefferson County (highest) and Lamar County (lowest) was 13.7% (Figure 2). Counties with higher obesity rates were clustered in the Mississippi Delta region and along the Mississippi River (Figure 2). Counties along the coastline and in the northeast region (Appalachian foothills) had lower obesity rates, as did counties with higher socioeconomic status, such as Madison and Rankin counties.

**Table:** 

**County**	**2009 Prevalence of Obesity, %**	**County**	**2009 Prevalence of Obesity, %**	**Quartiles, %**
Lamar	30.5	Simpson	35.5	30.5 - 33.3
Hancock	30.9	Yalobusha	35.6	33.4 - 35.5
DeSoto	30.9	Franklin	35.6	35.6 - 37.9
Rankin	31.1	Attala	35.9	38.0 - 44.2
Tishomingo	31.2	Leake	35.9	
Lafayette	31.6	Winston	35.9	
Itawamba	31.6	Benton	36.0	
Madison	31.9	Scott	36.2	
Alcorn	31.9	Grenada	36.4	
Lee	32.0	Wayne	36.5	
Union	32.1	Chickasaw	36.5	
Harrison	32.2	Montgomery	36.6	
Pearl River	32.4	Amite	36.8	
Pontotoc	32.4	Walthall	36.9	
George	32.5	Hinds	36.9	
Prentiss	32.6	Pike	37.0	
Jackson	32.7	Adams	37.2	
Tippah	33.1	Panola	37.4	
Webster	33.2	Marshall	37.7	
Neshoba	33.3	Clay	37.9	
Stone	33.3	Copiah	38.0	
Oktibbeha	33.3	Jasper	38.2	
Forrest	33.5	Yazoo	38.7	
Smith	33.6	Kemper	39.1	
Jones	33.8	Jefferson Davis	39.2	
Calhoun	34.0	Leflore	39.6	
Tate	34.4	Washington	39.6	
Newton	34.6	Bolivar	39.7	
Perry	34.6	Tallahatchie	39.9	
Greene	34.7	Issaquena	40.0	
Lauderdale	34.7	Sunflower	40.2	
Lincoln	34.8	Coahoma	40.5	
Monroe	34.9	Wilkinson	41.0	
Choctaw	35.1	Noxubee	41.0	
Lawrence	35.1	Sharkey	41.1	
Lowndes	35.2	Quitman	41.2	
Covington	35.2	Tunica	41.2	
Carroll	35.3	Humphreys	41.8	
Marion	35.4	Claiborne	42.0	
Warren	35.4	Holmes	42.7	
Clarke	35.5	Jefferson	44.2	

Respondents aged 30 to 44 years had the highest odds of being obese (Table 3). Compared with college graduates, respondents with an education level less than a bachelor's degree were more likely to be obese. Women had slightly higher odds than men, and blacks had much higher odds than whites. The interaction between sex and race was significant (*P* <.001); compared with white men, the odds of obesity were 2.2 (95% CI, 2.0-2.5) for black women, 1.4 (95% CI, 1.2-1.6) for black men, and 0.8 (95% CI, 0.7-0.9) for white women.

### Evaluation of county-level estimates

The width of 95% CIs for all county estimates was less than 5 percentage points (range 2.5-4.7 percentage points). This precision was comparable to that of the design-based direct estimates for the state and is considered reliable (25,26). To assess accuracy, we aggregated small-area county-level estimates to the state level for 2007 through 2009 and compared them with their respective state-level direct estimates; we also compared state-level model estimates with state-level direct estimates for 2007 through 2009. We further compared model estimates of age, sex, and race subgroups with their respective direct estimates. Pearson's correlation coefficients between the 2 sets of estimates for the comparisons were 0.95, 0.95, and 0.96, respectively, and the mean absolute differences were 0.5%, 0.5%, and 1.5%, respectively.

## Discussion

Using 2007-2009 BRFSS data, we obtained stable estimates for county-level prevalence of obesity. The 2009 obesity prevalence in all Mississippi counties is high: it shows the size and scope of the problem facing the state. This finding may help raise the awareness of the obesity crisis for state policy makers, health agency officials, journalists, and the general public. The county estimates also show that obesity prevalence among Mississippi counties varies considerably, which reveals the heterogeneous nature of the obesity epidemic in the state and requires targeted prevention measures to curb the trend. Furthermore, the escalating trend observed in Mississippi necessitates aggressive action. Mississippi has taken firm steps in confronting the child obesity crisis (27); parallel measures should be taken for adults.

Similar to observations reported by Li and colleagues (13), we found that in general, socioeconomic status was inversely related to the county prevalence of obesity among adults aged 18 years or older in Mississippi, after adjusting for age, sex, and race. This information may be helpful in guiding the direction of obesity prevention campaigns.

Confirming the troubling racial and socioeconomic disparities in obesity rates observed in other studies (2,3,28), our study shows that the risk of obesity for blacks nearly doubled that of whites, and black women had the highest odds of obesity. The 2000 Census indicates that Mississippi's population consists of 33.7% blacks, the highest in the nation (19). This means that compared with the rest of the nation, a larger proportion of Mississippi's population is in a higher risk category. Therefore, racial disparity in obesity affects Mississippi more than other states. The racial disparity, along with socioeconomic disparities, may influence public health efforts to plan and implement tailored prevention policies and programs because the effectiveness of health-promoting strategies varies among racial and socioeconomic groups. For instance, walking trails may be more effective in a community where a certain ethnic group clusters, and a farmer's market may be more effective in another community of different racial combinations. Consequently, prevention polices and programs would be most effective if designed accordingly.

This study has potential biases and limitations. First, because the weight and height values in BRFSS were self-reported, bias may have occurred (29,30). Compared with other surveys that generate estimates of obesity prevalence (eg, the National Health and Nutrition Examination Survey, for which trained professionals measure the height and weight of participants), BRFSS data tend to underestimate obesity prevalence. Some similar studies, attempting to minimize self-reporting bias, developed a correction method by using auxiliary data (13). However, since self-reporting bias affects all the county-level estimates similarly, it is not expected that relative distribution of obesity would vary. When estimates are biased, concerns arise about the magnitude of the bias. In the absence of an external standard against which to compare our data, our assessment of accuracy in this study is limited. Second, because of software limitations in handling standard error estimation when using mixed models for complex sample survey data, the 95% CI produced was a reasonable approximation, not exact. Further investigation in this direction may be needed.

## Figures and Tables

**Table 1:** Prevalence of Obesity,^a^ by County, BRFSS Mississippi, 2007

County	% (95% CI)
Lamar	28.7 (27.0-30.4)
Hancock	29.0 (27.5-30.6)
DeSoto	29.1 (27.3-30.9)
Rankin	29.2 (27.5-31.0)
Tishomingo	29.3 (27.7-31.0)
Lafayette	29.8 (28.0-31.6)
Itawamba	29.8 (28.1-31.5)
Madison	30.1 (27.9-32.3)
Alcorn	30.1 (28.5-31.8)
Lee	30.2 (28.4-32.0)
Union	30.2 (28.6-31.9)
Harrison	30.4 (28.8-32.1)
Pearl River	30.5 (28.9-32.2)
Pontotoc	30.5 (28.9-32.2)
George	30.6 (28.9-32.4)
Prentiss	30.7 (29.0-32.5)
Jackson	30.8 (29.2-32.4)
Tippah	31.2 (29.4-32.9)
Webster	31.3 (29.7-33.1)
Neshoba	31.4 (29.7-33.1)
Stone	31.4 (29.7-33.1)
Oktibbeha	31.4 (29.6-33.3)
Forrest	31.6 (29.9-33.4)
Smith	31.7 (30.0-33.4)
Jones	31.9 (30.2-33.6)
Calhoun	32.1 (30.4-33.8)
Tate	32.5 (30.9-34.2)
Newton	32.7 (31.0-34.5)
Perry	32.7 (30.9-34.6)
Greene	32.7 (30.8-34.7)
Lauderdale	32.8 (31.2-34.5)
Lincoln	32.8 (31.1-34.6)
Monroe	33.0 (31.3-34.8)
Choctaw	33.2 (31.4-35.0)
Lawrence	33.2 (31.5-34.9)
Lowndes	33.2 (31.6-34.9)
Covington	33.3 (31.6-35.0)
Carroll	33.4 (31.8-35.1)
Marion	33.4 (31.5-35.3)
Warren	33.4 (31.8-35.1)
Clarke	33.6 (31.9-35.3)
Simpson	33.6 (31.8-35.4)
Yalobusha	33.6 (32.0-35.3)
Franklin	33.7 (31.9-35.4)
Attala	33.9 (32.2-35.7)
Leake	33.9 (32.2-35.7)
Winston	34.0 (32.3-35.7)
Benton	34.1 (32.2-36.0)
Scott	34.3 (32.5-36.1)
Grenada	34.4 (32.6-36.2)
Wayne	34.5 (32.7-36.4)
Chickasaw	34.5 (32.7-36.4)
Montgomery	34.7 (33.0-36.5)
Amite	34.9 (33.1-36.6)
Walthall	34.9 (33.1-36.8)
Hinds	35.0 (33.2-36.8)
Pike	35.0 (33.3-36.8)
Adams	35.2 (33.6-36.9)
Panola	35.4 (33.6-37.3)
Marshall	35.7 (33.9-37.5)
Clay	35.9 (34.1-37.7)
Copiah	36.0 (34.1-37.9)
Jasper	36.2 (34.4-38.1)
Yazoo	36.7 (34.7-38.7)
Kemper	37.1 (35.1-39.1)
Jefferson Davis	37.2 (35.2-39.1)
Leflore	37.6 (35.7-39.6)
Washington	37.6 (35.8-39.5)
Bolivar	37.7 (35.7-39.7)
Tallahatchie	37.9 (35.7-40.0)
Issaquena	38.0 (35.8-40.2)
Sunflower	38.2 (36.0-40.3)
Coahoma	38.5 (36.5-40.5)
Wilkinson	38.9 (36.8-41.1)
Noxubee	39.0 (37.0-41.1)
Sharkey	39.0 (37.0-41.1)
Quitman	39.1 (37.0-41.3)
Tunica	39.2 (37.1-41.2)
Humphreys	39.7 (37.6-42.0)
Claiborne	40.0 (37.8-42.2)
Holmes	40.7 (38.5-42.9)
Jefferson	42.2 (39.8-44.6)

Abbreviations: BRFSS, Behavioral Risk Factor Surveillance System; CI, confidence interval.

a Body mass index ≥30.0 kg/m^2^ based on 2007 BRFSS self-reported data.

**Table 2:** Prevalence of Obesity^a^ Estimate, by County, BRFSS Mississippi, 2009

**County**	**% (95% CI)**
Lamar	30.5 (29.2-31.9)
Hancock	30.9 (29.6-32.1)
DeSoto	30.9 (29.4-32.4)
Rankin	31.1 (29.6-32.6)
Tishomingo	31.2 (29.9-32.5)
Lafayette	31.6 (30.2-33.1)
Itawamba	31.6 (30.3-33.0)
Madison	31.9 (29.9-34.0)
Alcorn	31.9 (30.7-33.2)
Lee	32.0 (30.6-33.4)
Union	32.1 (30.8-33.4)
Harrison	32.2 (31.0-33.6)
Pearl River	32.4 (31.1-33.7)
Pontotoc	32.4 (31.2-33.7)
George	32.5 (31.1-33.9)
Prentiss	32.6 (31.2-34.0)
Jackson	32.7 (31.4-33.9)
Tippah	33.1 (31.7-34.5)
Webster	33.2 (31.9-34.6)
Neshoba	33.3 (32.0-34.5)
Stone	33.3 (32.0-34.6)
Oktibbeha	33.3 (31.8-34.8)
Forrest	33.5 (32.2-34.9)
Smith	33.6 (32.3-34.9)
Jones	33.8 (32.5-35.1)
Calhoun	34.0 (32.7-35.3)
Tate	34.4 (33.2-35.7)
Newton	34.6 (33.3-36.0)
Perry	34.6 (33.1-36.2)
Greene	34.7 (33.0-36.3)
Lauderdale	34.7 (33.5-36.0)
Lincoln	34.8 (33.4-36.1)
Monroe	34.9 (33.6-36.3)
Choctaw	35.1 (33.7-36.5)
Lawrence	35.1 (33.8-36.4)
Lowndes	35.2 (33.9-36.5)
Covington	35.2 (33.9-36.5)
Carroll	35.3 (34.1-36.6)
Marion	35.4 (33.8-36.9)
Warren	35.4 (34.1-36.7)
Clarke	35.5 (34.2-36.9)
Simpson	35.5 (34.1-37.0)
Yalobusha	35.6 (34.3-36.9)
Franklin	35.6 (34.2-37.0)
Attala	35.9 (34.5-37.3)
Leake	35.9 (34.5-37.3)
Winston	35.9 (34.6-37.3)
Benton	36.0 (34.5-37.6)
Scott	36.2 (34.8-37.6)
Grenada	36.4 (35.0-37.8)
Wayne	36.5 (35.0-38.0)
Chickasaw	36.5 (35.1-38.0)
Montgomery	36.6 (35.3-38.0)
Amite	36.8 (35.5-38.2)
Walthall	36.9 (35.4-38.4)
Hinds	36.9 (35.5-38.5)
Pike	37.0 (35.6-38.4)
Adams	37.2 (35.9-38.5)
Panola	37.4 (35.9-38.9)
Marshall	37.7 (36.2-39.1)
Clay	37.9 (36.5-39.3)
Copiah	38.0 (36.4-39.6)
Jasper	38.2 (36.7-39.8)
Yazoo	38.7 (37.0-40.4)
Kemper	39.1 (37.4-40.8)
Jefferson Davis	39.2 (37.5-40.9)
Leflore	39.6 (37.9-41.3)
Washington	39.6 (38.1-41.2)
Bolivar	39.7 (38.0-41.4)
Tallahatchie	39.9 (38.0-41.8)
Issaquena	40.0 (38.0-42.0)
Sunflower	40.2 (38.3-42.1)
Coahoma	40.5 (38.8-42.2)
Wilkinson	41.0 (39.0-43.0)
Noxubee	41.0 (39.3-42.9)
Sharkey	41.1 (39.2-42.9)
Quitman	41.2 (39.2-43.1)
Tunica	41.2 (39.4-43.0)
Humphreys	41.8 (39.8-43.8)
Claiborne	42.0 (39.9-44.1)
Holmes	42.7 (40.7-44.8)
Jefferson	44.2 (41.9-46.6)

Abbreviations: BRFSS, Behavioral Risk Factor Surveillance System; CI, confidence interval.

a Body mass index ≥30.0 kg/m^2 ^based on 2009 BRFSS self-reported data.

**Table 3:** Odds of Obesity,^a^ by Socioeconomic Characteristics, Mississippi, 2007-2009

Characteristics	OR (95% CI)
**Age, y**	
18-29	1.00 [Reference]
30-44	1.77 (1.57-1.98
45-64	1.70 (1.52-1.90)
≥65	1.03 (0.90-1.17)
**Sex**	
Male	1.00 [Reference]
Female	1.18 (1.10-1.25)
**Race^b^**	
White alone or combined	1.00 [Reference]
Black alone or combined	1.88 (1.72-2.06)
**Education**	
Bachelor's degree or higher	1.00 [Reference]
Less than bachelor's degree	1.22 (1.12-1.33)
**Employment status**	
Employed	1.00 [Reference]
Unemployed	0.96 (0.89-1.04)
**Survey year**	
2007	1.00 [Reference]
2008	1.01 (0.94-1.09)
2009	1.10 (1.02-1.17)

Abbreviation: OR, odds ratio.

a Body mass index ≥30.0 kg/m^2 ^based on 2009 BRFSS self-reported data.

b Respondents who chose white alone or white with race(s) other than black were categorized as being in the white alone or combined category. Respondents who chose both black and white were categorized as black alone or combined. Respondents who chose only black or who chose black and other race(s) were categorized as being in the black alone or combined category.

## Appendix. Small Area Estimation Method Used to Calculate Obesity Prevalence by County, Mississippi, 2009

### First step

Let y_*ij*_ be the observation of obesity status, with *i* denoting county and *j* denoting individual respondent. Let X denote the design matrix for fixed-effect parameters *β* and their interaction terms; let Z denote the design matrix for random-effect parameters *γ* and their interaction terms. Then we have the mixed model:

Logit (y_*ij*_| *γ* ) = X*β* + Z*γ*

*γ *~ N (0, G)

where *γ* is assumed to be normally distributed with mean 0 and variance G. Let  and  denote the model-generated matrices that contain logits of fixed-effect and random-effect parameter estimates, respectively.  is general to every county, while  contains county-specific values.

### Second step

Let L represent the county-level fixed-effect coefficient matrix generated by using auxiliary data. The components in L correspond to the individual-level fixed-effect components in X. Then the logits of county-level prevalence of obesity estimates, , were calculated using formula (estimates in  were too small to be included):

= L

Next, an exponential transformation was applied to  to produce prevalence of obesity estimates  for each county:
